# LncRNA TUG1 attenuates ischaemia‐reperfusion‐induced apoptosis of renal tubular epithelial cells by sponging miR‐144‐3p via targeting Nrf2

**DOI:** 10.1111/jcmm.16924

**Published:** 2021-09-21

**Authors:** Sheng Zhao, Wu Chen, Wei Li, Weimin Yu, Siqi Li, Ting Rao, Yuan Ruan, Xiangjun Zhou, Cong Liu, Yucheng Qi, Fan Cheng

**Affiliations:** ^1^ Department of Urology Renmin Hospital of Wuhan University Wuhan China; ^2^ Department of Anesthesiology Renmin Hospital of Wuhan University Wuhan China

**Keywords:** apoptosis, LncRNA TUG1, miR‐144‐3p, Nrf2, renal ischaemia/reperfusion injury

## Abstract

Renal ischaemia/reperfusion (I/R) injury may induce kidney damage and dysfunction, in which oxidative stress and apoptosis play important roles. Long noncoding RNAs (lncRNAs) and microRNAs (miRNAs) are reported to be closely related to renal I/R, but the specific molecular mechanism is still unclear. The purpose of this research was to explore the regulatory effect of lncRNA TUG1 on oxidative stress and apoptosis in renal I/R injury. This research revealed that in renal I/R injury and hypoxia/reperfusion (H/R) injury in vitro, the expression level of lncRNA TUG1 was upregulated, and oxidative stress levels and apoptosis levels were negatively correlated with the expression level of lncRNA TUG1. Using bioinformatics databases such as TargetScan and microRNA.org, microRNA‐144‐3p (miR‐144‐3p) was predicted to be involved in the association between lncRNA TUG1 and Nrf2. This study confirmed that the level of miR‐144‐3p was significantly reduced following renal I/R injury and H/R injury in vitro, and miR‐144‐3p was determined to target Nrf2 and inhibit its expression. In addition, lncRNA TUG1 can reduce the inhibitory effect of miR‐144‐3p on Nrf2 by sponging miR‐144‐3p. In summary, our research shows that lncRNA TUG1 regulates oxidative stress and apoptosis during renal I/R injury through the miR‐144‐3p/Nrf2 axis, which may be a new treatment target for renal I/R injury.

## 1 INTRODUCTION

Renal ischaemia‐reperfusion injury (IRI), as a common type of clinical acute kidney injury (AKI), causes injury to the kidney and can even result in acute renal failure (ARF).^1^, ^2^ Convincing evidence has revealed the molecular and pathological events in AKI.^3^, ^4^, ^5^ Oxidative stress plays a vital role in renal IRI‐induced cell apoptosis and is the result of the imbalance between oxidative and antioxidant systems.^6^, ^7^ The production of excessive ROS eventually cause membrane lipid peroxidation and oxidative damage to proteins and DNA and lead to apoptosis and necrosis.^8^

MicroRNAs (miRNAs), small noncoding RNAs (ncRNAs), can regulate gene expression by targeting corresponding mRNAs. In the past ten years, there has been accumulating evidence showing that miRNAs play important roles in the development of various diseases, including renal IRI‐induced AKI.^9^, ^10^ Here, by RNA sequencing, we found that the expression of microRNA‐144‐3p was significantly changed in IRI‐induced kidneys. In addition, microRNA‐144‐3p has been reported to be closely related to oxidative damage in many studies. Therefore, we first studied the role of microRNA‐144‐3p in renal IRI.

However, long noncoding RNAs (lncRNAs) are a newly discovered group of RNA molecules that contain more than 200 nucleotides and represent important regulatory roles.^11^ LncRNAs control gene expression on multiple levels, including epigenetic, transcriptional, posttranscriptional and miRNA‐mediated mRNA transcription.^11^ According to recent studies, lncRNAs also play important roles in renal IRI.^12^, ^13^, ^14^, ^15^ LncRNA taurine upregulated gene 1 (TUG1), which plays important regulatory roles in models of oxidative damage induced by ischaemia,^16^, ^17^ was also reported to target miR‐144‐3p to play biological roles.^18^, ^19^ However, it is still unknown whether TUG1 may regulate apoptosis by targeting miR‐144 in renal IRI. To test this hypothesis, follow‐up experiments were conducted.

## 2 MATERIALS AND METHODS

### 2.1 Animal experiments and I/R model

Male C57BL/6 mice weighing 20–25 g (10–12 weeks of age) obtained from Hubei Center for Disease Control and Prevention were selected for animal experiments. All mice were kept in standard cages with free access to food and water. The room temperature was 18–25°C, and the humidity was controlled at 45–55%. All mice were bred normally for 1 week to adapt to environmental conditions and confirmed to be healthy according to the clinical examinations. All experiments were performed in accordance with the guidelines of the Ethics and Research Committee of Wuhan University Medical School (Wuhan, China).

A mouse model of renal IRI was established according to experimental requirements. In short, all mice were fasted overnight before surgery and anesthetized by an intraperitoneal injection of 1% pentobarbital sodium (0.3 ml/100 g). A midline abdominal incision was made to the mice in the IRI group, and the renal pedicle was dissected. Then, the bilateral renal pedicle was clamped with non‐traumatic clamps for 30 min. Subsequently, the non‐traumatic clamps were removed, and the kidney perfusion was restored for 48 h. Finally, the mice were euthanized by injecting excessive pentobarbital sodium, and their the kidney tissues were carefully collected for subsequent experiments. Mice in the sham operation group only received a median abdominal incision. Additionally, for transfection groups, miR‐144‐3p agomir (1 nmol/g/day), miR‐144‐3p antagomir (5 nmol/g/day), ASO‐TUG1 (5 nmol/g/day) and their negative controls (equal dosage) (RiboBio, Guangzhou, China) were injected from the tail vein for 3 consecutive days before the surgery. Blood samples were collected from individual mice to measure the concentration of serum creatinine.

### 2.2 Cell culture and the H/R model

Mouse renal tubular epithelial cells (TCMK cells) were purchased from Shanghai Institutes for Biological Sciences (Shanghai, China) and cultured in Dulbecco's modified Eagle's medium/nutrient mixture F‐12 (DMEM/F12) which was supplemented with 10% foetal bovine serum (FBS) (Hangzhou Sijiqing Biological Engineering Materials Company, Hangzhou, China), 100 µg/ml streptomycin and 100 U/ml penicillin. The cells were cultured in a humidified atmosphere containing 5% CO_2_ at 37°C. To mimic hypoxia reperfusion (H/R) injury in vitro, cells were cultured in an incubator filled with 1% O_2_, 5% CO_2_ and 94% N_2_ for different durations according to the experimental design and then subjected to reoxygenation for 6 h.

### 2.3 Cell transfection and reagents

The miR‐144‐3p mimic, miR‐144‐3p inhibitor and the corresponding negative control (miR‐NC), TUG1 smart silencer, Nrf2 siRNA and control siRNA (si‐NC) were all purchased from RiboBio (Guangzhou, China) and transfected into TCMK cells by using riboFECTTM CP Reagent following the manufacturer's instructions. For TUG1 overexpression in vitro, full‐length mouse TUG1 cDNA was amplified by PCR and then cloned into a pcDNA lentiviral vector (Hanheng, Shanghai, China). At the same time, Empty vector was used in the control group. Recombinant adenoviruses were packaged in 293T cells to generate lentiviruses. Forty‐eight hours after transfection, lentivirus was collected and incubated with TCMK cells overnight in the presence of polybrene. Finally, the cells were cultured in fresh complete growth medium for 48 h and then selected for another 10 days in medium containing puromycin (5 µg/ml). For all transfections, RT‐qPCR was used to verify transfection efficiency.

### 2.4 microRNA sequence

For microRNA sequencing, renal cortex of the left kidneys were obtained and TRIzol (Invitrogen) was used to extract total RNA, and microRNA sequencing was performed by Bioacme Biotechnology Company (Wuhan, China). Briefly, total RNA was purified with a ZR RNA microprep kit (Zymo Research) and randomly broken into short fragments. Then, a cDNA Library Construction Kit (TaKaRa, China) was used to generate cDNA libraries followed by purification, end repair, base A, joint sequencing and generation of cDNA fragments according to the manufacturer's instructions. After PCR amplification, microRNA sequencing was performed using the HiSeq 2500 sequencing platform (Illumina Company, San Diego, USA), which is high‐throughput and high‐sensitivity. For data analysis, differentially expressed miRNA profiles between the sham group and IR group were compared, and fold change and *p*‐value were calculated and used to identify significant differentially expressed miRNAs. Finally, Ingenuity Pathway Analysis software was used to investigate the functions and pathways involving differentially expressed microRNAs.

### 2.5 Luciferase report assay

TUG1 luciferase reporter constructs were constructed by inserting wild‐type or mutant TUG1 sequence containing miR‐144‐3p binding site into the downstream of psiCHECK reporter plasmid, and named them TUG1‐Wt and TUG1‐Mut, respectively. A similar method was used to produce Nrf2‐Wt and Nrf2‐Mut. Subsequently, miR‐144‐3p or miR‐NC and luciferase reporter constructs were co‐transfected into TCMK cells using Lipofectamine 2000 (Invitrogen, USA). Finally, Dual‐Luciferase Reporter Assay System (Promega, Madison, WI, USA) was used to analyse luciferase activity after 48 h of transfection.

### 2.6 RNA pulldown assay and RNA immunoprecipitation (RIP) assay

RNA pulldown assays were performed to examine the interaction between TUG1 and miR‐144‐3p. Biotin RNA Labeling Mix was used for Biotin labelling according to the manufacturer's instructions. TUG1 containing either the Wt or Mut binding sequence was cloned. RNeasy Mini Kits (Qiagen, Valencia, CA) were used to purify TUG1 according to the manufacturer's protocol. Then, the probe‐coated beads were generated with the incubation of the biotinylated TUG1 probe and nanobeads. TCMK cells were then incubated for 24 h, and RT‐qPCR analyses were performed after eluting the RNA complex from the beads.

RIP assays were performed using the Magna RIPTM RNA‐Binding Protein Immunoprecipitation Kit (17‐700; Millipore, USA) according to the manufacturer's instructions. TCMK cells were first treated with RIP lysis buffer and then incubated with RIP buffer containing magnetic beads conjugated with anti‐AGO2 antibodies (1:200, ab186733; Abcam) or anti‐IgG (Millipore). Finally, RT‐qPCR was performed to determine the enrichment of TUG1. IgG was used as a negative control to normalize RNA‐IPs.

### 2.7 Real‐time quantitative polymerase chain reaction (RT‐qPCR)

Total RNA was extracted from TCMK cells and the cortex of left kidneys with TRIzol reagent following the manufacturer's instructions. For the detection of lncRNA, total RNA was reverse‐transcribed into cDNA by the reverse transcription kit (RR047A, Takara, Japan). Furthermore, for the detection of miRNA, the tailed method was applied, and the NCode^TM^ miRNA First‐Strand cDNA Synthesis Kit (MIRC10, Invitrogen) was employed to polyadenylate the isolated RNA. The samples were loaded following which the samples were subjected to reverse transcription quantitative polymerase chain reaction (RT‐qPCR) with the 7900 HT RT‐ PCR System (Applied Biosystems, USA). Three replicate wells were set per sample to obtain variable data. Additionally, GAPDH or U6 were used as an internal reference. The following primer sequences were used in the study: miR‐144‐3p, forward 5’‐GCTG GGATATCATCATATACTG‐3’ and reverse 5’‐CGGACTAGTACATCATCTATACTG‐3’; TUG1, forward 5’‐GGCACCCAGTGTAAAGCA‐3’ and reverse 5’‐AAGCAGCAGATAACAGAGTTG A‐3’; GAPDH, forward 5’‐GTCAACGGATTTGGTCTGTATT‐3’ and reverse 5’‐AGTCTTCTGG GTGGCAGT‐3’; U6, forward 5’‐CTCGCTTCGGCAGCACATA‐3’ and reverse 5’‐AACGATTC ACGAATTTGCGT‐3’.

### 2.8 Flow cytometry (FCM) analysis

To assess the level of apoptosis, Annexin V‐fluorescein‐5‐isothiocyanate (Annexin V‐FITC) apoptosis detection kit (BD Pharmingen, Beijing, USA) was used to analyse phosphatidylserine exposure according to the manufacturer's protocol. To make it short, TCMK cells in different groups were collected and washed two times with cold phosphate‐buffered saline (PBS). Then, the washed cells were centrifuged and resuspended in 500 µl 1× binding buffer, and incubated with 5 µl Annexin and 5 µl propidium iodide (PI) for about 15 min in the dark. Finally, the cells were analysed by FCM (Becton Dickinson, San Jose, CA, USA), and then, CELL Quest 3.0 software (Becton Dickinson) was used for data analysis.

### 2.9 Detection of MDA and SOD in kidney tissue or cellular supernatant

MDA and SOD levels were detected to assess oxidative stress level in the kidney tissue and cell samples. The kidney tissue samples were weighed, and Tris buffer (pH 7.4) was added according to the weight. Then, the tissue samples were homogenized and centrifuged at 12,500 g for 20 min at 4°C. The supernatants were collected after centrifugation. Subsequently, the xanthine oxidase method and TBA method were used to detect the SOD and MDA content in the samples, respectively. Finally, automatic microplate reader (Multiskan MK3, Thermo Scientific) was used to detect absorbance. SOD levels were expressed as U/mg protein in tissue and U/ml in cell supernatants, and MDA levels were expressed as nmol/mg protein in tissue and nmol/ml in cell supernatants.

### 2.10 TUNEL detection

In situ apoptosis detection kit (Promega) was used to detect the level of apoptosis in different tissues. Observed under a light microscope, the nuclei of apoptotic cells were stained brown, while the nuclei of negative cells were stained blue. The apoptosis rate was calculated based on the percentage of positive cell nuclei, which were stained brown in the visual field. The cell count under the microscope was independently completed by two professional pathologists in a blind manner.

### 2.11 Haematoxylin and eosin (HE) staining

The obtained kidney tissues were fixed in 4% formalin, dehydrated and embedded in paraffin. 5‐μm‐thick paraffin sections were cut out for subsequent staining. The sections were deparaffinized and stained with haematoxylin and eosin. Kidney damage was divided into the following grades according to HE staining: 0 (absence of necrosis), 1 (mild necrosis), 3 (moderate necrosis) and 5 (severe necrosis).

### 2.12 Immunohistochemical (IHC) staining

The obtained kidney tissues were fixed in formalin. The fixed samples were embedded in paraffin and cut into 4‐µm‐thick sections, then deparaffinized, rehydrated, and blocked. Sections were first incubated with the primary antibodies rabbit anti‐mouse cleaved‐caspase3 (1:300, ab2302, Abcam) or rabbit anti‐mouse NGAL (1:200, ab216462, Abcam) at 4°C overnight. The sections were then washed three times with PBS and incubated with horseradish peroxidase (HRP)‐conjugated IgG for 30 min, and then incubated with 3,3'‐diaminobenzidine (DAB). Finally, the sections were counterstained with haematoxylin. The observation under the microscope was performed by two independent professional pathologists. The NGAL expression score was graded as follows: 0 (no staining), 1 (weak but detectable staining), 2 (distinct staining) and 3 (intense staining).

### 2.13 Immunofluorescence (IFC) staining

Cryosections (10 µm) of mouse kidneys were fixed in 4% paraformaldehyde. After the sections were washed with PBS, they were incubated with primary antibody against Nrf2 (1:300, ab137550; Abcam) at 4°C overnight. Then, sections were incubated with appropriate fluorescein‐labelled IgG for 1 h. Finally, sections were washed with PBS, and nuclei were stained blue with 5 µg/ml (4’,6‐diamidino‐2‐phenylindole; Beyotime, Nantong, China) DAPI for 2 min. A fluorescence microscope (Nikon 80i, Japan) was used to assess Nrf2 expression.

### 2.14 Transmission electron microscopy

Transmission electron microscopy (TEM) was conducted to observe possible mitochondrial morphological changes induced by IRI in vivo. Briefly, sesame‐sized kidney cortex tissues from each group were fixed in 2.5% paraformaldehyde at 4°C for 24 h and then treated with 2% osmium tetroxide at 4°C for 2 h. After multiple rinse cycles with double‐distilled water, the samples were dehydrated in a graded series of acetone and then embedded in Spurr's resin for ultrathin sectioning. Ultrathin sections were cut to 50‐nm thickness on an ultramicrotome using diamond knives. Subsequently, the sections were stained with 2% uranyl acetate and citrate at 25°C for 1 h. Finally, the sections were visualized under a transmission electron microscope.

### 2.15 Plasma creatinine detection

Mouse blood samples for testing were obtained through cardiac puncture and accumulated in a tube. Plasma creatinine was obtained after centrifugation at 3000 rpm at 4°C for 10 min; then, the sarcosine oxidase method with a commercial creatinine assay kit (Nanjing Jiancheng Bioengineering Institute, Nanjing, China) was used to detect the samples. Creatinine levels were expressed as mg/dl.

### 2.16 Western blot analysis

Proteins from kidney tissue samples or TCMK cells were obtained using RIPA lysis buffer (Solarbio, Beijing, China) containing 0.1 mM PMSF (Beyotime Institute of Biotechnology, Haimen, China) and a protease inhibitor (Roche). For Cyt c detection, cytosolic and mitochondrial extracts were collected using a mitochondrial extraction kit (Beyotime Institute of Biotechnology, Haimen, China) according to the manufacturer's instructions. Protein samples in each group (40 µg/lane) were subjected to 10% or 15% SDS‐PAGE and then transferred to PVDF membrane at 200 mA. Then, the membrane was blocked for 1 h and incubated with primary antibody overnight at 4°C. The primary antibodies used were as follows: Bax (PA5‐70418, 1:2000, Thermo Fisher Scientific); Bcl‐2 (PA5‐68611, 1:2000; Thermo Fisher Scientific); Cyt c (NB100‐56503, 1:5000; Novus Biologicals); cleaved caspase‐3 (1:3000, ab2302; Abcam); CHOP (NB600‐1335, 1:2000; Novus Biologicals); GRP78 (ab108615, 1:1000; Abcam); Nrf2 (MAB3925, 1:1000; Novus Biologicals); HO‐1 (ab13243, 1:2000, Abcam); and β‐actin (ab28052, 1:5000; Abcam). After washing 3 times with TBST, the membrane was incubated with anti‐mouse/rabbit secondary antibody (P/N 925‐32210, P/N 925‐32211, 1:10,000; LI‐COR Biosciences) for 1 h at 37°C.

Finally, a Western blot detection system (LI‐COR Biosciences, Lincoln, NE, USA) was used to scan the bands, and Odyssey v1.2 software (LI‐COR Biosciences) was used for semi‐quantitative analysis of protein expression.

### 2.17 Statistical analysis

All experimental data are presented as the means ± standard deviation (*SD*). Each group of experiments was repeated at least three times or each group contained at least six mice. The statistical software package SPSS version 19.0 was used for statistical analysis. Differences between two groups were compared using two‐side unpaired t test, while the comparisons between multiple groups were performed using one‐way analysis of variance (ANOVA) with Tukey's post hoc test. *p* < 0.05 was considered significant.

## 3 RESULTS

### 3.1 MiR‐144‐3p was downregulated in I/R‐injured kidney tissue in vivo and H/R‐challenged TCMK cells in vitro

**FIGURE 1:**
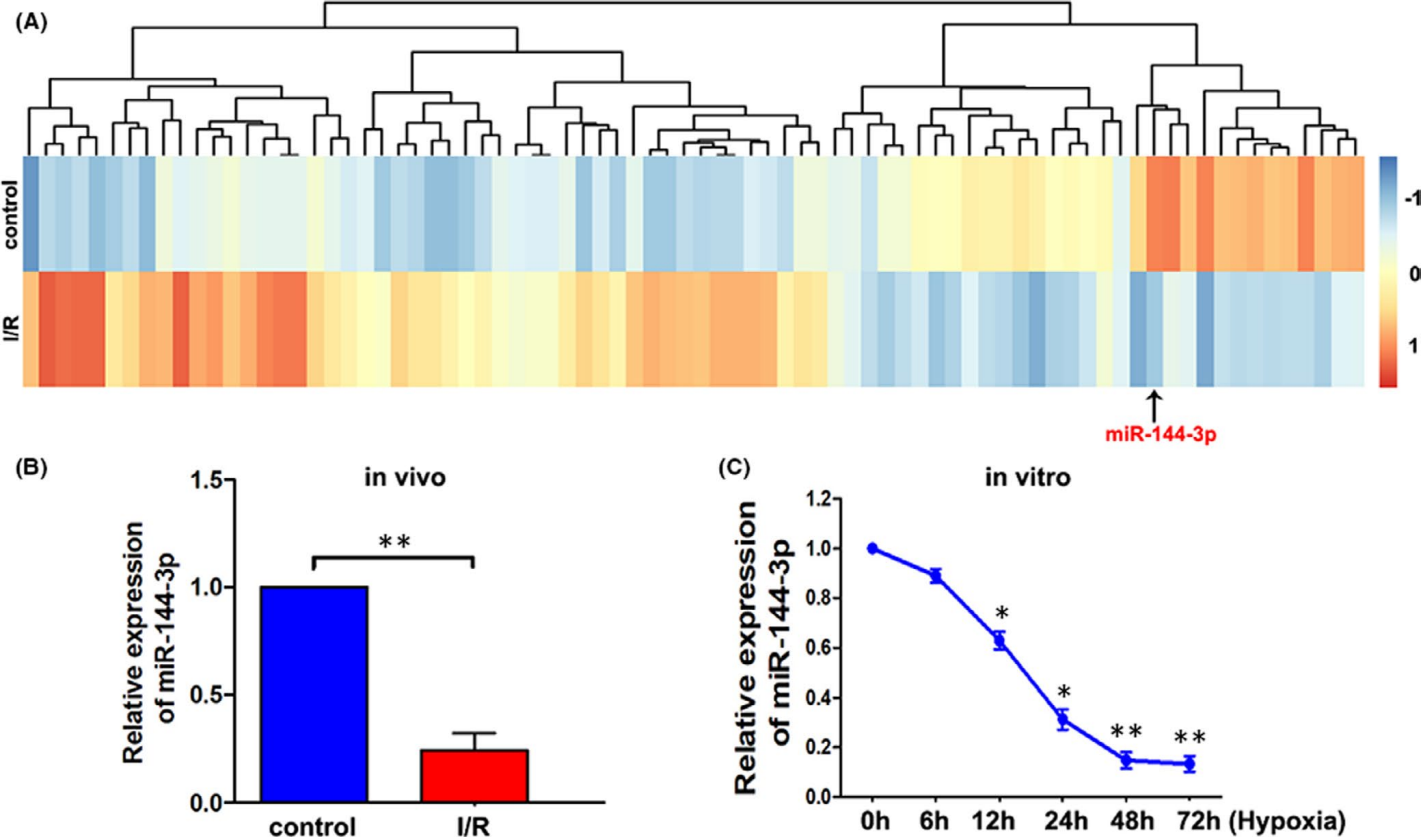
MiR‐144‐3p was downregulated in ischemia reperfusion‐injured kidney tissue in vivo and hypoxia reperfusion‐challenged TCMK cells in vitro. A, IIIumina's Solexa sequencing technology was used to analyze the differentially expressed miRNAs between I/R‐injured and control kidney samples in mice. B, The level of miR‐144‐3p was measured by RT‐qPCR in normal and I/R‐injured kidney tissue in vivo (*n* = 3). C, The level of miR‐144‐3p in TCMK cells under different hypoxia times (all the groups reperfused for the same time, 6 h) was detected by RT‐qPCR (*n* = 3). Differences between two groups were compared using unpaired *t* test. *p < 0.05, **p < 0.01

To identify which microRNAs are differentially expressed during kidney ischaemia/reperfusion injury, we performed RNA‐sequencing analysis of I/R‐injured kidney tissue compared with control kidney tissue from C57BL/6 mice. A total of 80 differentially expressed miRNAs with at least twofold changes and their *p*‐values were all less than 0.05. As shown in Figure 1A, the heat map indicated differentially expressed miRNAs and the relative expression levels identified by microarray assay were displayed. Then, the level of miR‐144‐3p was further validated by real‐time PCR assay, and the results showed that miR‐144‐3p levels were significantly lower in I/R‐injured kidney tissues than in normal tissues (Figure 1B). To further determine the function of miR‐144‐3p in the I/R‐injured kidney model, we further investigated miR‐144‐3p expression by RT‐PCR assay in a cellular hypoxia reperfusion model using TCMK cells. As shown in Figure 3C, the cells were deprived of oxygen (1% O2) for 0, 6, 12, 24, 48 and 72 h (all the groups were reperfused for the same time, 6 h). MiR‐144‐3p levels significantly decreased when the hypoxia time reached 12 h and continuously decreased to approximately 20% at 48 h of hypoxia. No obvious decrease was observed when the hypoxia time continued to increase to 72 h. Therefore, we set the hypoxia time at 48 h for subsequent cell experiments.

### 3.2 Nrf2 was a direct target of miR‐144‐3p

**FIGURE 2:**
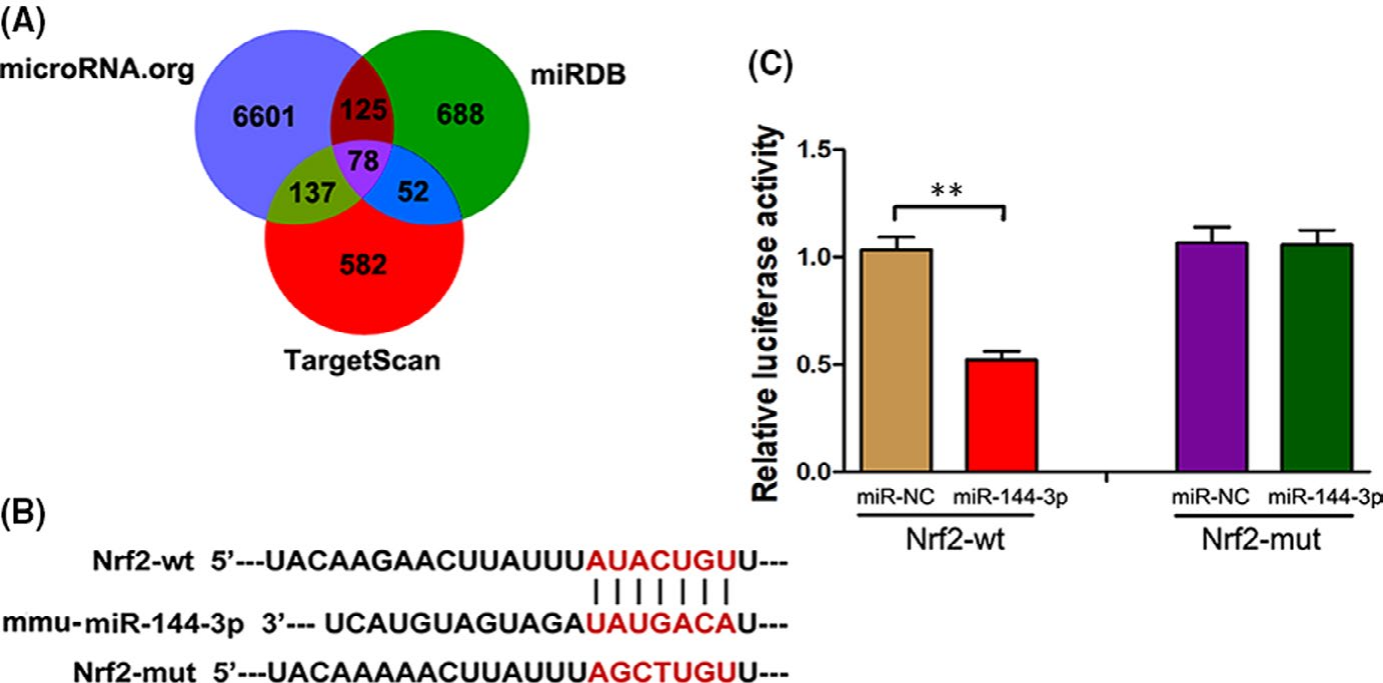
Nrf2 was a direct target of miR‐144‐3p. A, A schematic diagram of searching target genes of miR‐144‐3p in three databases. B, A schematic diagram showing the potential binding site between miR‐144‐3p and Nrf2. C, After luciferase construct and miR‐144‐3p mimic or miR‐NC were co‐transfected into TCMK cells, the relative luciferase activity was measured in Nrf2‐Wt group and Nrf2‐mut group (*n* = 3). All data are expressed as the mean ± *SD*; data comparisons between multiple groups were performed using one‐way analysis of variance (ANOVA) with Tukey's post hoc test. **p < 0.01

TargetScan (http://www.targetscan.org/), microRNA.org (http://www.microrna.org/) and miRDB (http://www.mirdb.org) were used to predict the target genes of miR‐144‐3p. The results in Figure 2A revealed that miR‐144‐3p can regulate multiple target genes and that Nrf2 was one of them. TargetScan software was first applied to identified the potential binding site of the mouse Nrf2 gene for mmu‐miR‐144‐3p (Figure 2B). Next, the luciferase construct containing the Wt or mut binding sequence of Nrf2 was co‐transfected into TCMK cells with the miR‐144‐3p mimic or miR‐NC. The Luciferase report assay was subsequently carried out, and the results showed that the miR‐144‐3p mimic suppressed luciferase expression containing the WT‐Nrf2 sequence but not the Mut‐Nrf2 sequence (Figure 2C). The above results all showed that Nrf2 was a direct target of miR‐144‐3p in TCMK cells.

### 3.3 Influence of miR‐144‐3p on H/R‐induced renal tubular epithelial cell apoptosis in vitro

**FIGURE 3:**
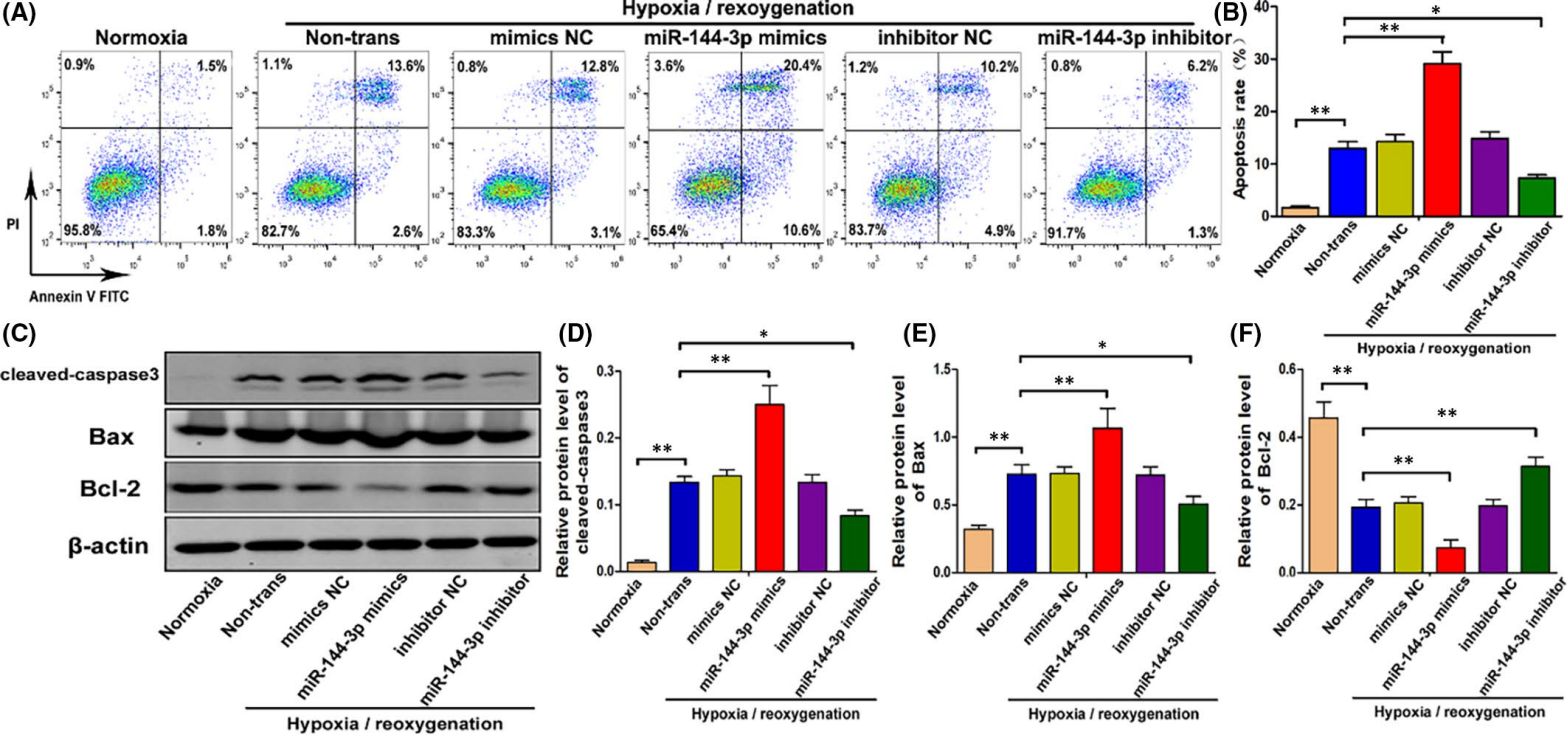
MiR‐144‐3p mimics alleviates H/R‐induced apoptosis in vitro. A, B, Cell apoptosis of normal TCMK cells and cells transfected with miR‐144‐3p mimics, miR‐144‐3p inhibitor or their corresponding negative control was examined by flow cytometry after H/R treatment (*n* = 3). C–F, The expression level of cleaved caspase3, Bax and Bcl‐2 in normal TCMK cells and cells transfected with miR‐144‐3p mimics, miR‐144‐3p inhibitor or their corresponding negative control was measured by Western blot after H/R treatment. Protein bands are shown in C, and the quantitative analysis of protein expression level is shown in D–F (*n* = 3). All data are expressed as the mean ± *SD*; data comparisons between multiple groups were performed using one‐way analysis of variance (ANOVA) with Tukey's post hoc test. *p < 0.05, **p < 0.01

MiR‐144‐3p, which plays an important role in many diseases, can induce cell apoptosis by regulating oxidative damage. Our experiments confirmed that Nrf2, a key gene that regulates oxidative damage, is one of the direct targets of miR‐144‐3p. Therefore, it was reasonable to speculate that miR‐144‐3p could change the degree of oxidative stress and regulate cell apoptosis by targeting Nrf2. First, to explore the specific role of miR‐144‐3p in H/R‐induced renal tubular epithelial cell apoptosis, miR‐144‐3p mimics, miR‐144‐3p inhibitor and their corresponding negative control group (mimics NC, inhibitor NC) were transfected into TCMK cells. Flow cytometry in Figure 3A,B shows that H/R injury unsurprisingly increased cell apoptosis; transfection with miR‐144‐3p mimics markedly aggravated cell apoptosis compared with the mimics NC group; transfection with miR‐144‐3p inhibitor effectively reduced cell apoptosis compared with the inhibitor NC group. Furthermore, Western blot analysis showed that transfection with miR‐144‐3p mimics increased the expression level of cleaved‐caspase3 and Bax and decreased the expression level of Bcl‐2. Consistent with this result, transfection with the miR‐144‐3p inhibitor obviously decreased the expression level of proapoptotic proteins and increased the expression level of Bcl‐2. In short, the above results indicated that miR‐144‐3p accelerated H/R‐induced apoptosis in TCMK cells.

### 3.4 MiR‐144‐3p regulates H/R‐induced Nrf2‐HO‐1 signalling pathway activation, oxidative stress, mitochondria and endoplasmic reticulum functions in vitro

**FIGURE 4:**
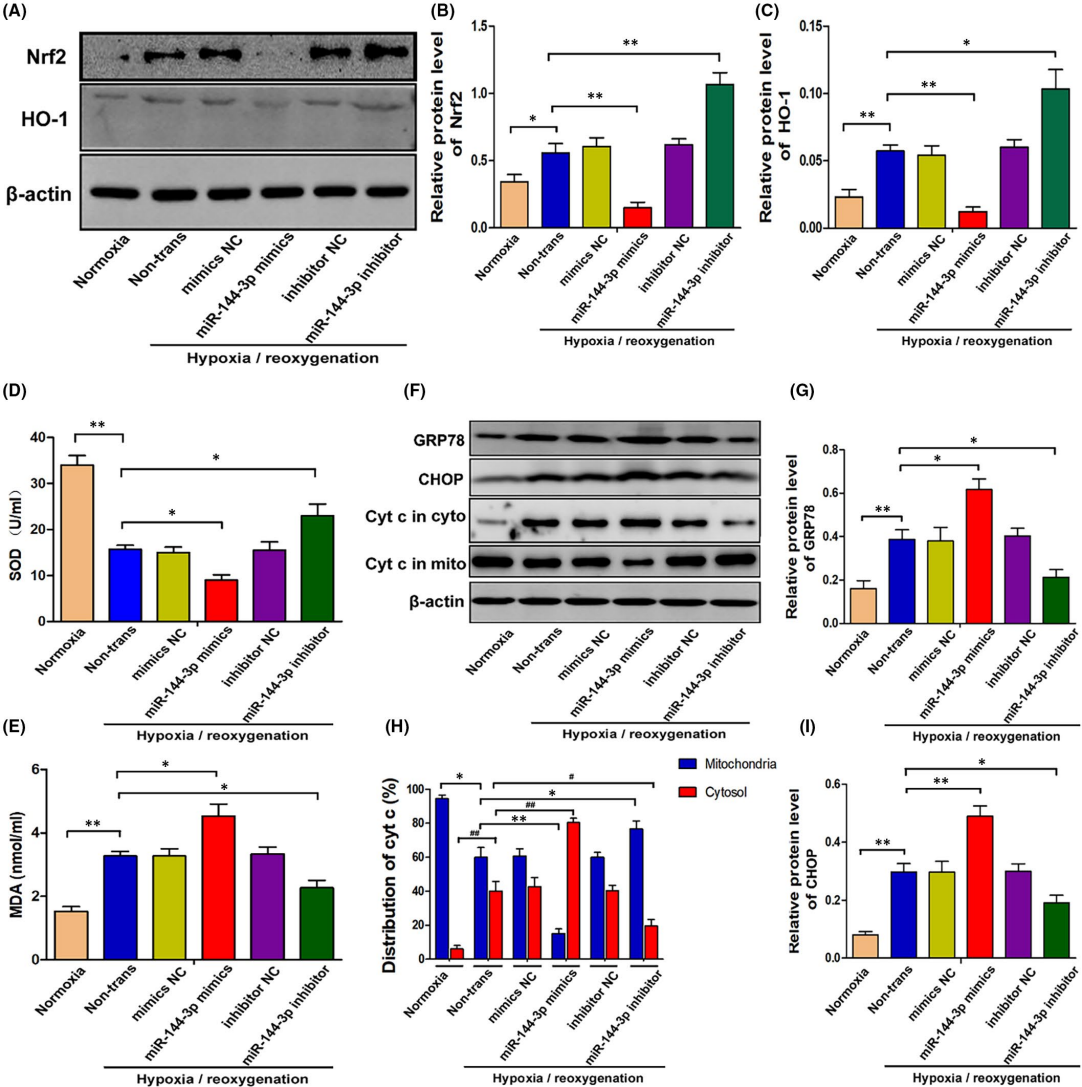
MiR‐144‐3p regulates H/R‐induced Nrf2‐HO‐1 signaling pathway activation, oxidative stress, mitochondria and endoplasmic reticulum functions in vitro. A–C, The expression levels of Nrf2 and HO‐1 in TCMK cells transfected with miR‐144‐3p mimics or inhibitor after H/R treatment. Representative bands are shown in A, and the quantitative analysis of protein expression level is shown in B, C (*n* = 3). D, E, The SOD and MDA levels in cellular supernatant of TCMK cells transfected with miR‐144‐3p mimics or inhibitor after H/R treatment (*n* = 3). F–I, The expression levels of CHOP, GRP78 and Cytochrome C in TCMK cells transfected with miR‐144‐3p mimics or inhibitor after H/R treatment. Representative bands are shown in F, and the quantitative analysis of protein expression level is shown in G–I (*n* = 3). All data are expressed as the mean ± *SD*; data comparisons between multiple groups were performed using one‐way analysis of variance (ANOVA) with Tukey's post hoc test. *p < 0.05, **p < 0.01

As mentioned above, Nrf2 was a confirmed target of miR‐144‐3p. However, the Nrf2‐HO‐1 signalling pathway plays an important role in regulating oxidative stress, and increased levels of oxidative stress can eventually induce apoptosis by altering the function of mitochondria and the endoplasmic reticulum. As shown in Figure 4A–C, the expression of Nrf2 and its downstream signalling molecule HO‐1 were clearly downregulated when transfected with miR‐144‐3p mimics and upregulated when transfected with miR‐144‐3p inhibitor in H/R‐induced TCMK cells. To detect the oxidative stress level in TCMK cells, SOD and MDA levels were measured in cellular supernatants (Figure 4D,E). The results indicated that SOD levels were decreased and MDA levels were increased when transfected with miR‐144‐3p mimics. In contrast, SOD levels were increased and MDA levels were decreased when transfected with miR‐144‐3p inhibitor. Finally, the expression levels of Cyt c, CHOP and GRP78, which are representative molecules of mitochondria and endoplasmic reticulum functions, were assessed. As shown in Figure 4F–I, miR‐144‐3p mimics effectively increased the expression levels of CHOP, GRP78 and cytochrome C in the cytoplasm but decreased the level of cytochrome C in mitochondria. However, the miR‐144‐3p inhibitor effectively decreased the expression levels of CHOP, GRP78 and cytochrome C in the cytoplasm but increased the level of cytochrome C in mitochondria. Therefore, these findings revealed that miR‐144‐3p could regulate oxidative stress, mitochondria and endoplasmic reticulum functions in H/R‐induced TCMK cells and Nrf2‐HO‐1 signalling pathway was closely involved.

### 3.5 MiR‐144‐3p regulates I/R‐induced kidney damage probably though adjusting Nrf2 expression, oxidative stress and mitochondrial damage in vivo

**FIGURE 5:**
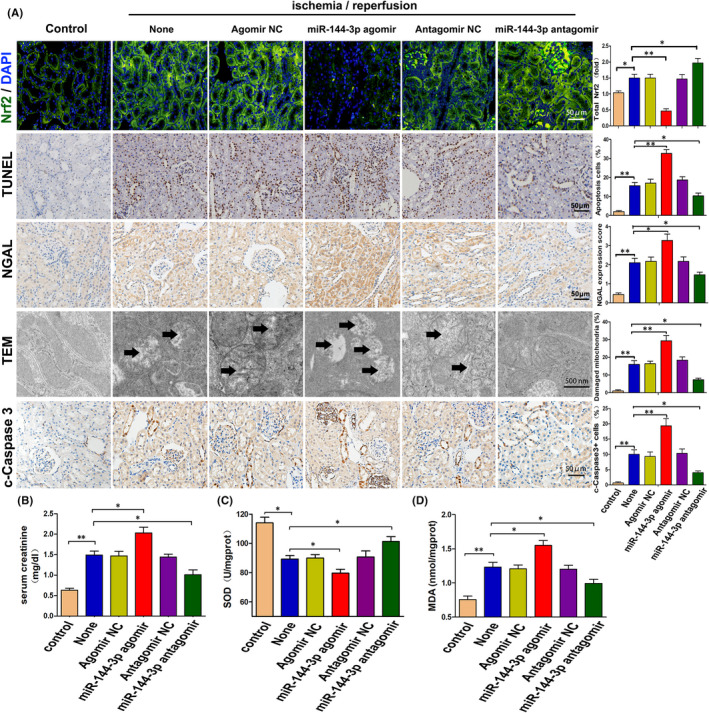
MiR‐144‐3p regulates I/R‐induced kidney damage probably though adjusting Nrf2 expression, oxidative stress and mitochondrial damage in vivo. A, Representative images and respective quantification of Nrf2 expression via immunofluorescence, TUNEL staining, NGAL and cleaved‐caspase3 expression via immunohistochemical staining, and mitochondrial structure by TEM in mouse kidneys with different treatments. The black arrows show swollen or vacuolar mitochondria. Data were obtained from 3 images per mouse, with 6 mice per group. B, Levels of serum creatinine in mice with miR‐144‐3p agomir or miR‐144‐3p antagomir treatments (*n* = 6). C, D, SOD and MDA levels in kidney tissue with miR‐144‐3p agomir or miR‐144‐3p antagomir treatments (*n* = 6). All data are expressed as the mean ± *SD*; data comparisons between multiple groups were performed using one‐way analysis of variance (ANOVA) with Tukey's post hoc test. *p < 0.05, **p < 0.01

To further determine the role of miR‐144‐3p in I/R‐induced kidney damage, we established kidney I/R models after tail intravenous injections of miR‐144‐3p agomir, miR‐144‐3p antagomir and their corresponding negative control groups. As shown in Figure 5, we first assessed kidney Nrf2 expression in different groups via immunofluorescence. The results showed that the expression of Nrf2 was clearly downregulated with the injection of miR‐144‐3p agomir and upregulated with the injection of miR‐144‐3p antagomir (Figure 5A). In addition, the results of the TUNEL assay and cleaved‐caspase3 immunohistochemical examination (Figure 5A) showed that I/R‐induced cell apoptosis was markedly aggravated by the injection of miR‐144‐3p agomir but significantly reduced by the injection of miR‐144‐3p antagomir. We also detected the expression of NGAL in kidney tissue via immunohistochemical staining (Figure 5A) and creatinine levels in serum (Figure 5B) to detect kidney damage overall. The results indicated an aggravation of kidney damage with miR‐144‐3p agomir treatment and an alleviation of kidney damage with miR‐144‐3p antagomir treatment. Moreover, similar results were observed when oxidative stress and mitochondrial damage were compared between the nontrans group and the miR‐144‐3p agomir group or miR‐144‐3p antagomir group. Oxidative stress was evaluated by the detection of SOD and MDA in kidney tissue (Figure 5C,D), and mitochondrial damage was assessed by transmission electron microscopy.

### 3.6 Knocking down Nrf2 reversed the miR‐144‐3p inhibitor‐induced alleviation of H/R‐induced apoptosis, oxidative stress, mitochondrial damage and endoplasmic reticulum stress in vitro

**FIGURE 6:**
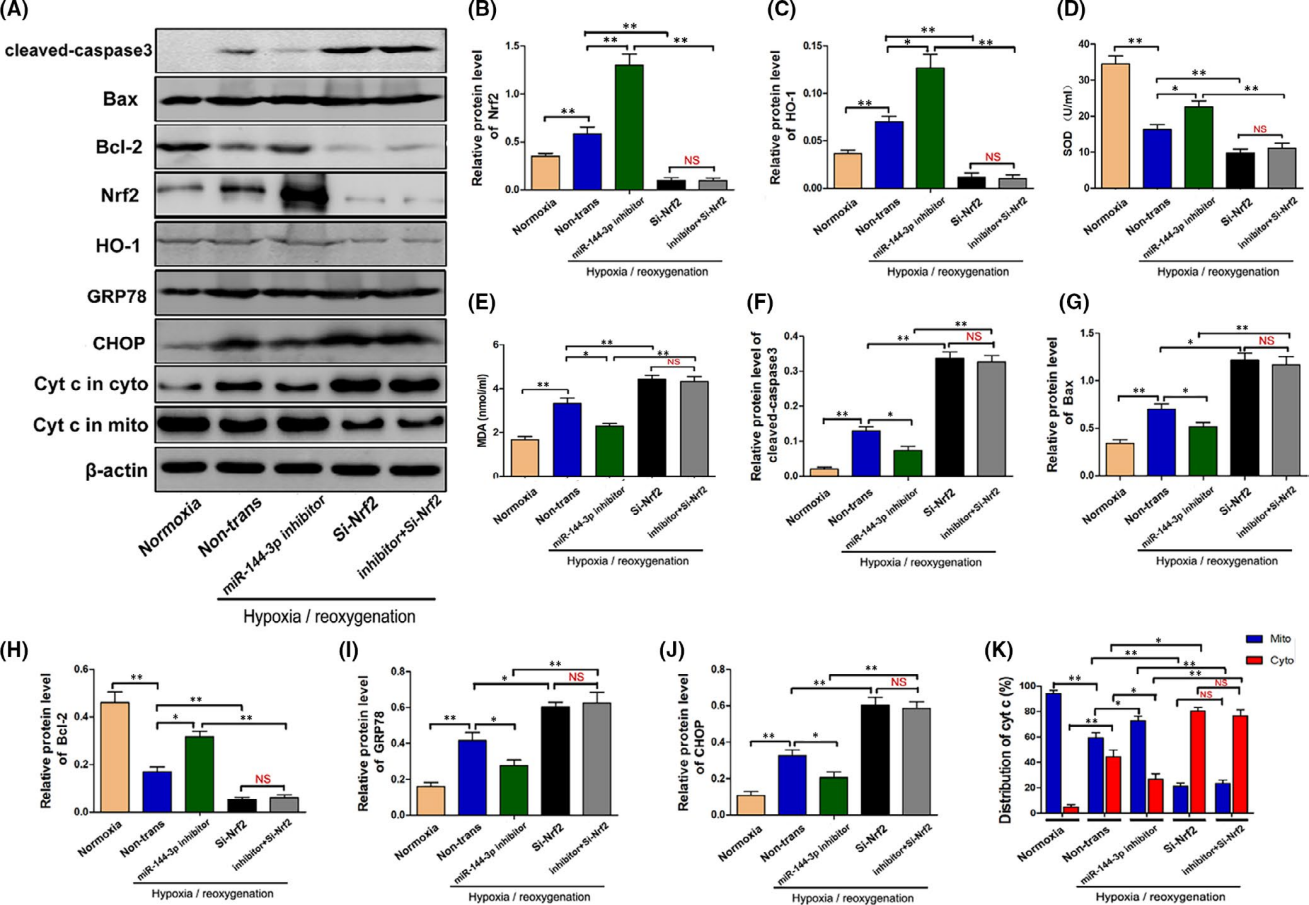
Knocking down Nrf2 reversed the miR‐144‐3p inhibitor‐alleviated H/R‐induced apoptosis, oxidative stress, mitochondrial damage and endoplasmic reticulum stress in vitro. A–c, F–K, The protein expression level of Nrf2, HO‐1, cleaved‐caspase3, Bax, Bcl‐2, CHOP, GRP78 and Cytochrome C in H/R‐injured TCMK cells transfected with miR‐144‐3p inhibitor or si‐Nrf2. Representative bands are shown in A, and the quantitative analysis of protein expression is shown in B, C, F–K (*n* = 3). D, E, The SOD and MDA levels in cellular supernatants in H/R‐injured TCMK cells transfected with miR‐144‐3p inhibitor or si‐Nrf2 (*n* = 3). All data are expressed as the mean ± *SD*; data comparisons between multiple groups were performed using one‐way analysis of variance (ANOVA) with Tukey's post hoc test. *p < 0.05, **p < 0.01, NS, no significant difference

To verify the important protective effect of Nrf2 in miR‐144‐3p–inhibited cell apoptosis during H/R injury, miR‐144‐3p inhibitor and si‐Nrf2 were co‐transfected into TCMK cells before hypoxia treatments. The expression level of Nrf2 and HO‐1 was clearly upregulated when transfected with miR‐144‐3p inhibitor and distinctly downregulated when transfected with siNrf2 or miR‐144‐3p inhibitor and siNrf2 co‐transfected. The difference was not statistically significant when comparing the siNrf2 group with the miR‐144‐3p inhibitor and siNrf2 co‐transfected group (Figure 6A–C). Next, we detected the SOD and MDA levels in cellular supernatants to evaluate oxidative stress. The results in Figure 6D,E illustrate that the miR‐144‐3p inhibitor effectively reduced oxidative stress level by enhancing the level of SOD and reducing the level of MDA. However, the protective effect was reversed when siNrf2 was co‐transfected. Similar results were observed when cell apoptosis, mitochondrial damage and endoplasmic reticulum stress were evaluated, as shown in Figure 6A, F–K. The result of Western blot analysis showed that the miR‐144‐3p inhibitor effectively suppressed the expression levels of cleaved caspase‐3, Bax, CHOP, GRP78 and cytochrome C in the cytoplasm but increased the levels of Bcl‐2 and cytochrome C in mitochondria. These protein expression levels were reversed when si‐Nrf2 was co‐transfected at the same time. Moreover, siNrf2 alone could obviously change the above indicators, and no significant differences were observed when compared with the miR‐144‐3p inhibitor group and siNrf2 co‐transfected group (Figure 6A, F–K). In short, the above results indicated that miR‐144‐3p regulates H/R‐induced apoptosis, oxidative stress, mitochondrial damage and endoplasmic reticulum stress by adjusting the Nrf2‐HO‐1 signalling pathway.

### 3.7 TUG1 was upregulated in kidney IRI in vivo and in vitro, and miR‐144‐3p was a direct target of TUG1

**FIGURE 7:**
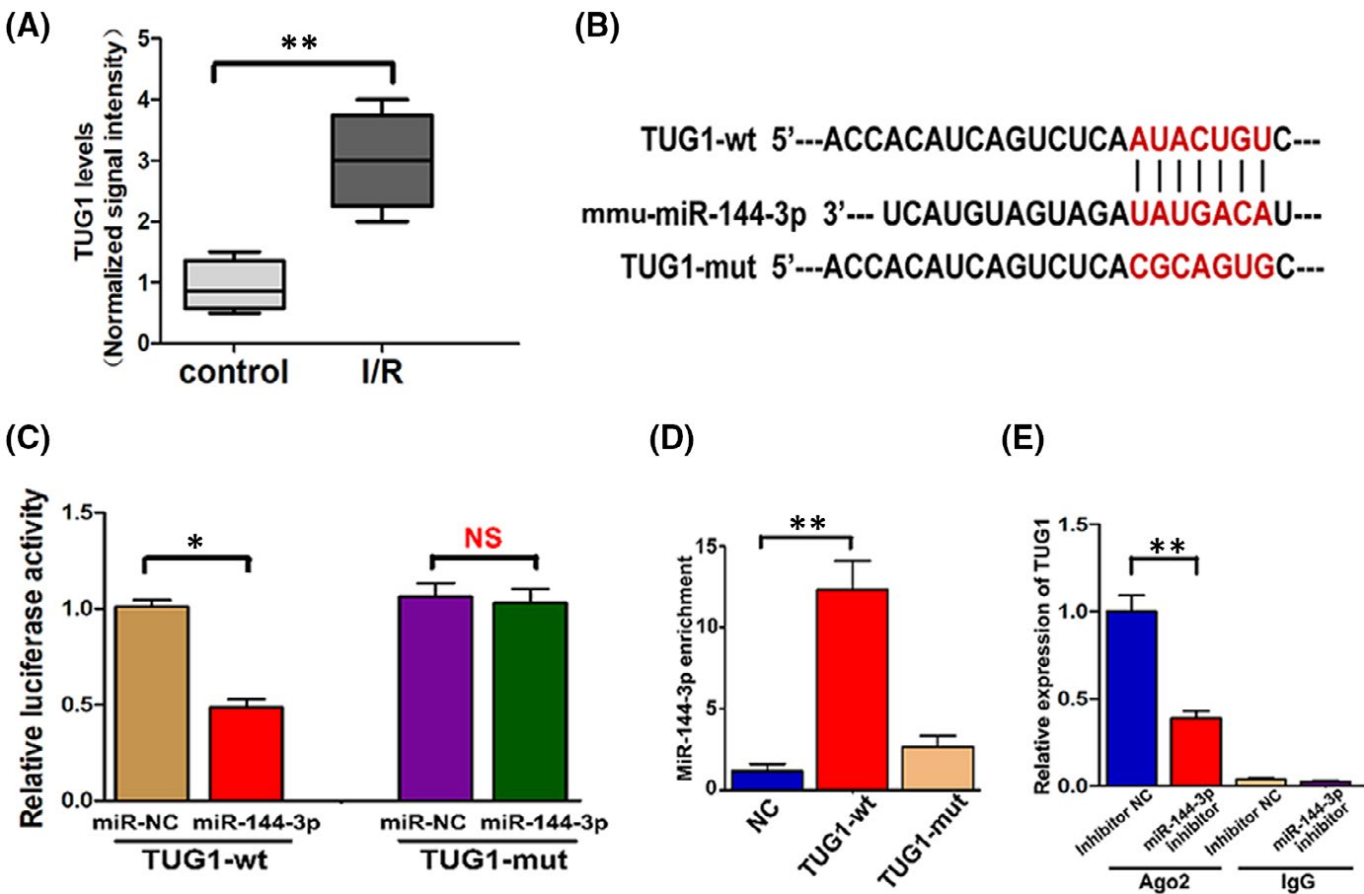
TUG1 was upregulated in kidney tissues during renal IRI, and miR‐144‐3p was a direct target of TUG1. A, The TUG1 level was measured in normal and I/R‐injured kidney tissues by RT‐qPCR (*n* = 6). B, A schematic diagram showing the potential binding site between TUG1 and miR‐144‐3p. C, After luciferase construct and miR‐144‐3p mimic or miR‐NC were co‐transfected into TCMK cells, the relative luciferase activity was measured in TUG1‐Wt group and TUG1‐mut group (*n* = 3). D, RNA pulldown analysis using TUG1‐Wt showed that TUG1 directly interacted with miR‐144‐3p in TCMK cells, and TUG1‐Mut failed to pull down miR‐144‐3p (*n* = 3). E, After RIP, RT‐qPCR was used to detect the total amount of TUG1 bound to Ago2 or IgG (*n* = 3). All data are expressed as the mean ± *SD*. Differences between two groups were compared using unpaired *t* test. Data comparisons between multiple groups were performed using one‐way analysis of variance (ANOVA) with Tukey's post hoc test. *p < 0.05, **p < 0.01, NS, no significant difference

It is known the lncRNA TUG1 can alleviate oxidative stress level and inhibit apoptosis in many diseases. In addition, miR‐144‐3p is reported to be a direct target of TUG1. To explore whether TUG1 also plays an important role in renal tubular epithelial cells during IRI, the expression of TUG1 in vivo and in vitro were measured by real‐time PCR assay. In vivo, TUG1 in I/R‐injured kidney tissues was significantly upregulated compared to normal tissues (Figure 7A). To confirm whether miR‐144‐3p was a direct target of TUG1, TargetScan was used to identify the potential binding site of TUG1 for mmu‐miR‐144‐3p (Figure 7B). After the luciferase construct containing the Wt or mut binding sequence of TUG1 was co‐transfected into TCMK cells with the miR‐144‐3p mimics or miR‐NC, the luciferase reporter assay was performed and results showed that miR‐144‐3p mimics significantly reduced the luciferase activity in TUG1‐Wt group (Figure 7C). Luciferase report assay revealed that miR‐144‐3p was a direct target of TUG1. Moreover, the result of RNA pulldown assay indicated that miR‐144‐3p directly interacted with TUG1‐Wt but not TUG1‐mut, indicating that the interaction between TUG1 and miR‐144‐3p is sequence‐specific (Figure 7D). Finally, the results of RIP assay showed that TUG1 was detected in Ago2 immunoprecipitates in control group. In contrast, the expression of TUG1 was significantly reduced in Ago2 complexes purified from cell samples transfected with miR‐144‐3p inhibitor, indicating that TUG1 is likely in the miR‐144‐3p RISC complex (Figure 7E). In summary, the above results indicated that TUG1 was upregulated in kidney during renal IRI and that miR‐144‐3p was directly regulated by TUG1 in TCMK cells.

### 3.8 TUG1 plays an important role in H/R‐induced cell apoptosis possibly through regulating the Nrf2‐HO‐1 pathway, oxidative stress, mitochondrial damage and endoplasmic reticulum stress via targeting miR‐144‐3p

**FIGURE 8:**
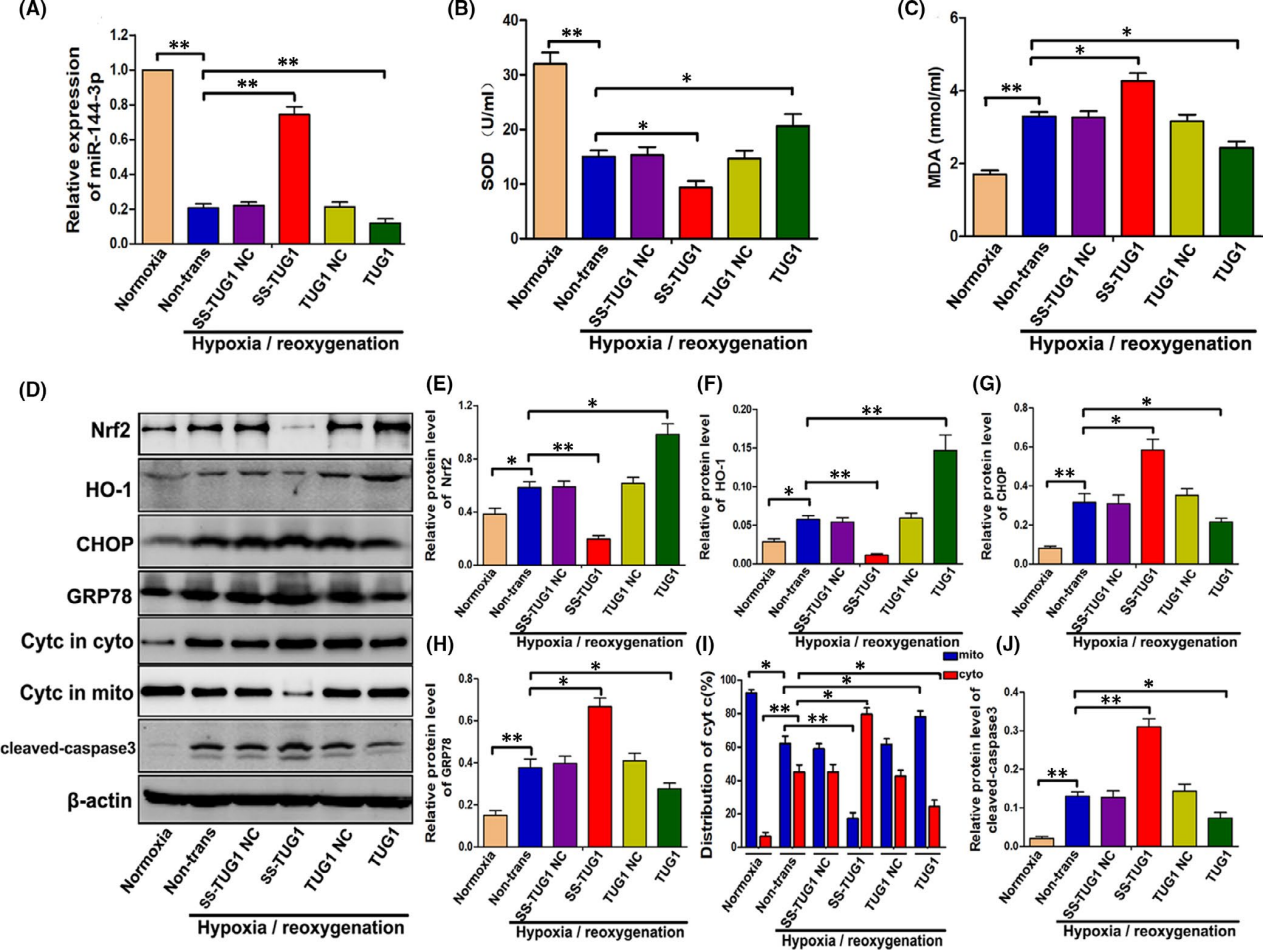
TUG1 plays an important role in H/R‐induced cell apoptosis possibly through regulating the Nrf2‐HO‐1 pathway, oxidative stress, mitochondrial damage and endoplasmic reticulum stress via targeting miR‐144‐3p. A, The expression of miR‐144‐3p in TUG1‐overexpressing or TUG1‐knockdown TCMK cells detected by RT‐qPCR (*n* = 3). B, C, The levels of SOD and MDA in cellular supernatant (*n* = 3). D–J, The relative expression levels of Nrf2, HO‐1, GRP78, CHOP, Cyt C and cleaved‐caspase3 were examined. Representative protein bands are shown in D, and the quantitative analysis of protein expression is shown in E‐J (*n* = 3). All data are expressed as the mean ± *SD*; data comparisons between multiple groups were performed using one‐way analysis of variance (ANOVA) with Tukey's post hoc test. *p < 0.05, **p < 0.01

To evaluate the specific effect of TUG1 in TCMK cells after H/R treatment, we overexpressed TUG1 via transfection of recombinant adenoviruses and knocked down TUG1 expression via transfection of TUG1 smart silencer, which consisted of three small interfering RNA (siRNA) sequences and three antisense oligonucleotide (ASO) sequences. As a direct target of TUG1, the level of miR‐144‐3p expression was then detected. Figure 8A shows that TUG1 knockdown increased miR‐144‐3p expression and that TUG1 overexpression decreased miR‐144‐3p expression. Immediately thereafter, we assessed the activation of the Nrf2‐HO‐1 pathway, degree of oxidative stress, mitochondrial damage and endoplasmic reticulum stress. As shown in Figure 8B,C, the levels of SOD were decreased and levels of MDA were increased when TUG1 was knocked down; in contrast, the levels of SOD were increased and the levels of MDA were decreased when TUG1 was overexpressed. In addition, TUG1 knockdown effectively increased the expression levels of cleaved caspase3, CHOP, GRP78 and cytochrome C in the cytoplasm but suppressed the expression levels of Nrf2, HO‐1 and cytochrome C in mitochondria (Figure 8D–J). However, TUG1 overexpression significantly suppressed the expression of cleaved caspase‐3, CHOP, GRP78 and cytochrome C in the cytoplasm but increased the expression levels of Nrf2, HO‐1 and cytochrome C in mitochondria (Figure 8D–J). In summary, our data suggested that TUG1 could possibly influence H/R‐induced cell apoptosis through regulating the Nrf2‐HO‐1 pathway, oxidative stress, mitochondrial damage and endoplasmic reticulum stress via targeting miR‐144‐3p.

### 3.9 TUG1 knockdown aggravated I/R‐injured kidney damage possibly through adjusting Nrf2 expression, oxidative stress and mitochondrial damage in vivo

**FIGURE 9:**
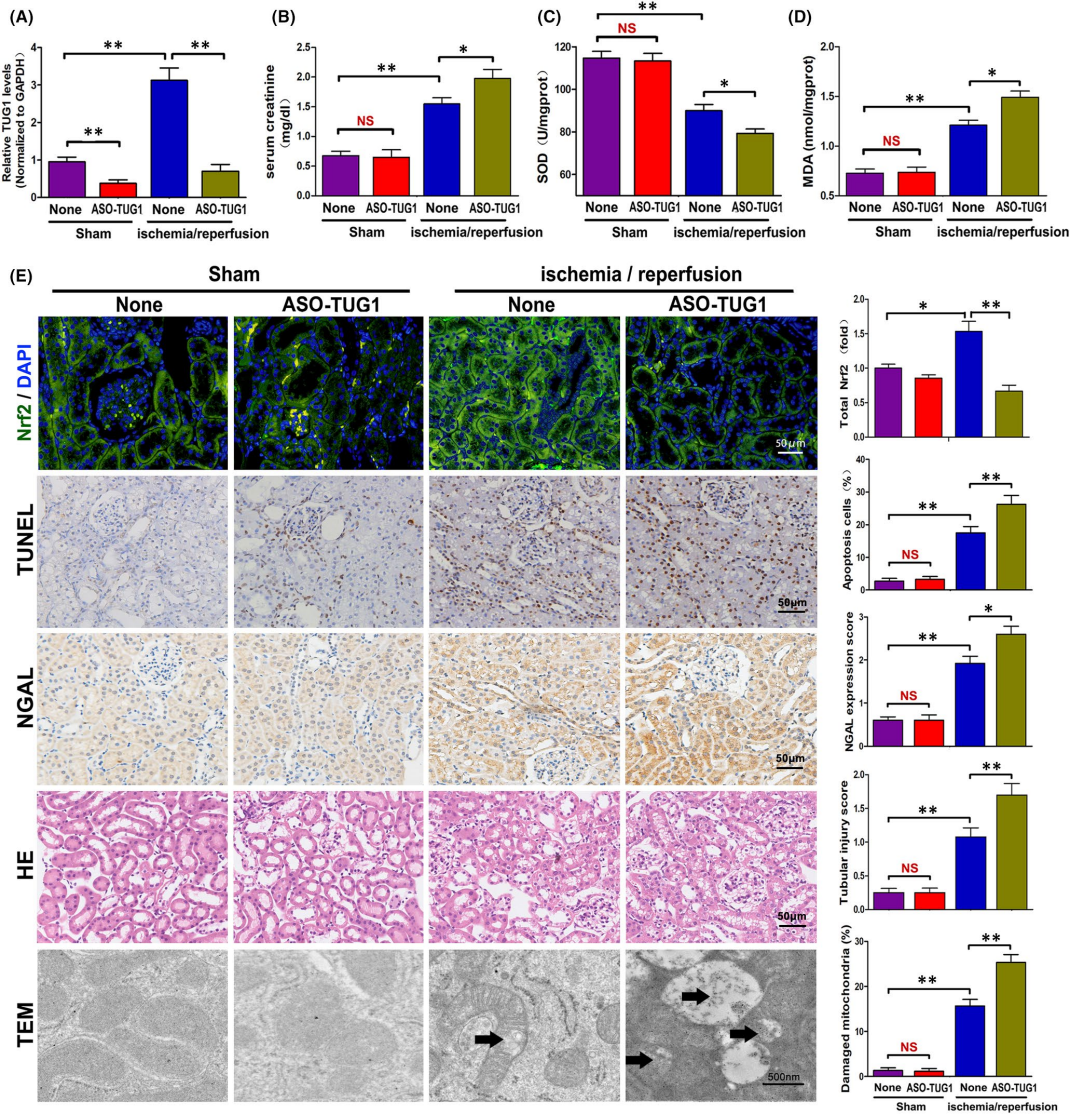
TUG1 knockdown aggravated I/R‐injured kidney damage by adjusting Nrf2 expression, oxidative stress and mitochondrial damage in vivo. A, Expression of TUG1 in mouse kidneys detected by RT‐qPCR (*n* = 6). B, Levels of serum creatinine in mice treated with TUG1‐ASO (*n* = 6). C, D, SOD and MDA Levels in kidney tissue with TUG1‐ASO treatments (*n* = 6). E, Representative images and respective quantification of Nrf2 expression via immunofluorescence, TUNEL staining, NGAL expression via immunohistochemical staining, HE staining and mitochondrial structure by TEM in TUG1‐ASO‐treated mouse kidneys. The black arrows show swollen or vacuolar mitochondria. Data were obtained from three images per mouse, with six mice per group. All data are expressed as the mean ± *SD*; data comparisons between multiple groups were performed using one‐way analysis of variance (ANOVA) with Tukey's post hoc test. *p < 0.05, **p < 0.01, NS, no significant difference

To demonstrate the specific effect of TUG1 in vivo, we knocked down the expression of TUG1 in mice by tail intravenous injection of TUG1 antisense oligonucleotide (ASO). The downregulation of TUG1 was confirmed in mouse kidneys by RT‐qPCR (Figure 9A). Immunofluorescence figures displayed a markedly reduced expression of Nrf2 with the injection of ASO acid‐TUG1 in I/R‐injured kidneys. TUNEL staining, NAGL expression via immunohistochemical staining and HE staining, as well as serum creatinine, were examined to evaluate kidney damage overall. The results showed significantly aggravated kidney injury with the injection of ASO‐TUG1 (Figure 9B, E). Then, oxidative stress and mitochondrial damage were appraised by SOD and MDA detection and TEM. Unsurprisingly, the levels of SOD and MDA and the number of damaged mitochondria were apparently increased in I/R‐injured kidneys with ASO‐TUG1 injection. Otherwise, no significant difference was observed in the above indicators between the sham operation groups with or without ASO‐TUG1 injection (Figure 9C–E). In summary, TUG1 knockdown aggravated I/R‐injured kidney damage, which may be attributed to upregulated oxidative stress and mitochondrial damage adjusted through Nrf2 downregulation.

### 3.10 The TUG1‐miR‐144‐3p‐Nrf2 axis regulates H/R‐induced cell apoptosis by adjusting oxidative stress and endoplasmic reticulum stress in vitro

**FIGURE 10:**
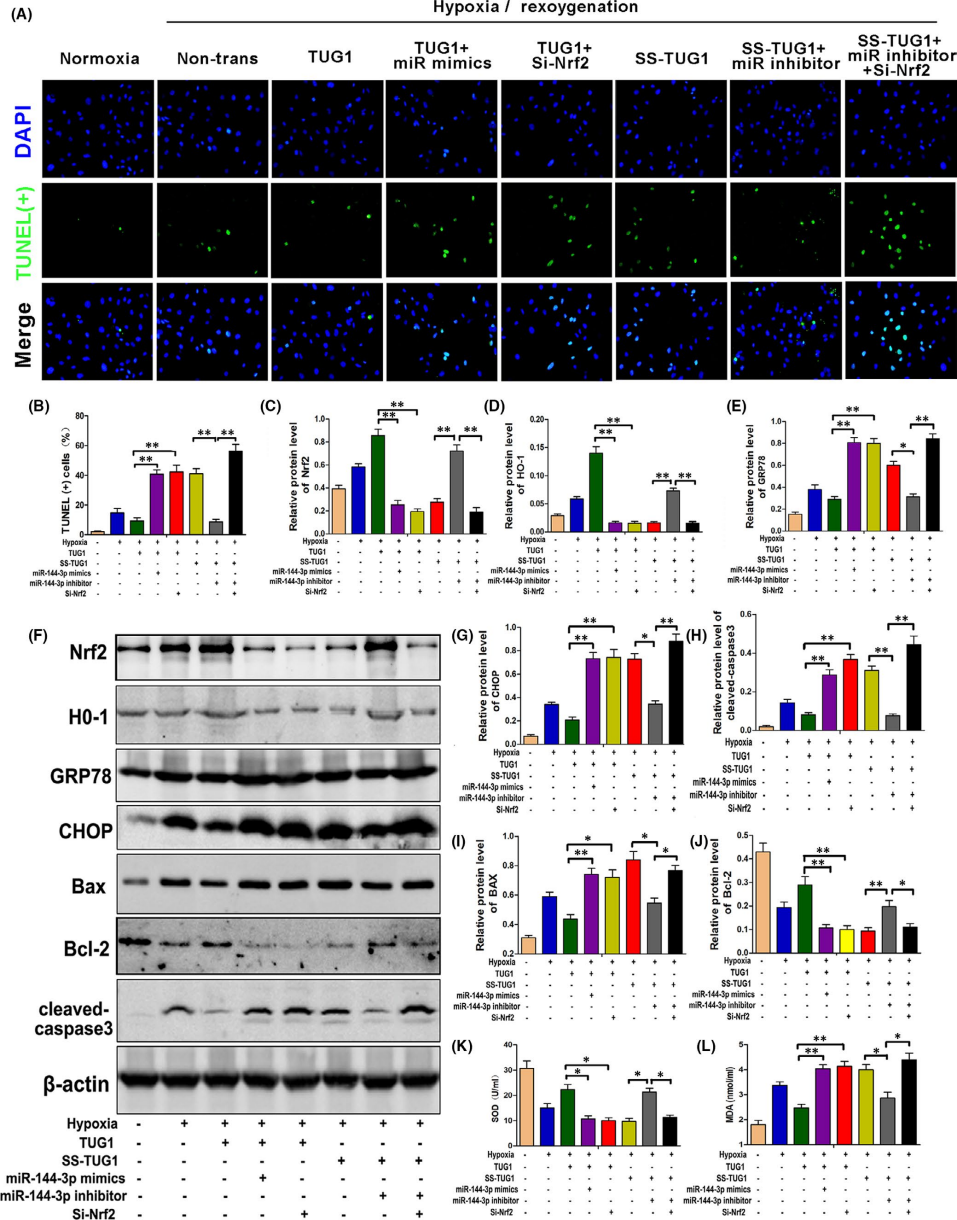
The TUG1‐miR‐144‐3p‐Nrf2 axis regulates H/R‐induced cell apoptosis by adjusting oxidative stress and endoplasmic reticulum stress in vitro. A, B, TUNEL stain and quantitative analysis for H/R‐injured TCMK cells with different transfections (*n* = 3). C–J, The expression levels of Nrf2, H0‐1, CHOP, GRP78, Bax, Bcl‐2 and cleaved‐caspase3 in H/R‐injured TCMK cells with different transfections were measured by Western blot analysis. Representative protein bands are shown in F, and the quantitative analysis of protein expression is shown in C‐E, G‐J (*n* = 3). K, L, The levels of SOD and MDA in different transfection groups (*n* = 3). All data are expressed as the mean ± *SD*; data comparisons between multiple groups were performed using one‐way analysis of variance (ANOVA) with Tukey's post hoc test. *p < 0.05, **p < 0.01

According to our data, the opposite regulation and functional roles of TUG1 and miR‐144‐3p in I/R‐ or H/R‐induced TCMK cells verified that TUG1, by targeting miR‐144‐3p, suppressed oxidative stress, mitochondrial damage and endoplasmic reticulum stress‐triggered cell death by adjusting the Nrf2‐HO‐1 pathway. To explore the specific role of miR‐144‐3p or Nrf2 in the TUG1‐miR‐144‐3p‐Nrf2 axis, further experiments were performed. Based on Western blot analysis and SOD and MDA detection, we found that TUG1 overexpression reduced cell apoptosis by adjusting the Nrf2‐HO‐1 pathway, oxidative stress and endoplasmic reticulum stress, which could be reversed by transfected with si‐Nrf2 or miR‐144‐3p mimics. However, TUG1 knockdown exacerbated cell apoptosis, which could be also reversed by transfected with the miR‐144‐3p inhibitor. However, this protective treatment could be reversed again once Nrf2 was knocked down at the same time (Figure 10).

## 4 DISCUSSION

Renal IR is a common phenomenon in clinical operations such as organ procurement, vascular surgery and renal transplantation. Furthermore, In addition, renal IRI is one of the main clinical causes of ARF and is accompanied by high mortality.^20^, ^21^ Therefore, it is clear that it is very necessary to understand the specific molecular mechanism of renal IRI and explore its treatment methods.

In recent years, accumulating researches have revealed that miRNAs are closely related to the pathogenesis and progression of renal IRI.^22^, ^23^, ^24^ In order to clarify the changes of microRNAs systematically and comprehensively during renal IRI, RNA‐sequencing analysis was firstly performed. Among all these obviously changed miRNAs, miR‐144‐3p was preliminarily selected for further studies because it was never studied in renal IRI. Subsequent RT‐qPCR detection also confirmed that miR‐144‐3p was significantly downregulated in I/R‐injured kidney tissue in vivo and H/R‐challenged TCMK cells in vitro. Overexpression of miR‐144‐3p was previously reported to induced apoptosis in multiple myeloma by targeting c‐Met.^25^ Similarly, our experiments demonstrated that the expression of miR‐144‐3p in renal tubular cells was closely related to cell apoptosis. Therefore, it is reasonable to speculate that miR‐144‐3p is a key regulatory factor in renal IRI.

MicroRNAs were widely confirmed to perform functions by inhibiting protein translation via binding to their target mRNA. Nrf2, as an extensively studied gene, was demonstrated to play key roles in oxidative damage related disease including renal IRI.^26^, ^27^, ^28^ Besides, several previously studies had already confirmed that Nrf2 was a potential target of miR‐144‐3p.^29^, ^30^ However, the potential relationship between Nrf2 and miR‐144‐3p was never studied in a renal IRI model. In our study, Nrf2 was selected Nrf2 as a downstream target gene of miR‐144‐3p for subsequent experiments.

Nrf2 is a modular protein with seven Nrf2‐ECH homology domains (Neh1–7), each of which fulfills distinct functions^31^ and the transcriptional activity and spatial distribution of Nrf2 have a greater impact on its function.^32^ As a negative regulator of Nrf2,^33^ Keap1 is a substrate adaptor protein for the ubiquitin ligase Cul3/Rbx1, which mediates the degradation of Nrf2 by interacting with DLG and ETGE motifs on the Neh2 domain of Nrf2.^34^ Nrf2 maintains at a relatively low level in the cytoplasm. However, in stressed situations like IRI, oxidative stress occurs and Keap1 is oxidized at reactive cysteine residues and inactivated.^35^ As a result, Nrf2 is released from Keap1, and its intracellular expression level has also been improved. Subsequently, Nrf2 enters the nucleus and binds to the sMaf protein to form a heterodimer,^36^ which is combined with the antioxidant response element (ARE 5′‐TGACXXXGC‐3′) in the promoter sequence of the target genes of Nrf2 to promote the expression of its target genes,^37^ and HO‐1 is one of them.^38^ With the activation of the Nrf2/HO‐1 pathway, the expression levels of antioxidant proteins such as SOD increases, thereby reducing oxidative stress, improving the function of mitochondria and endoplasmic reticulum, and reducing apoptosis, and this is completely consistent with our experiments.

Consistent with previous studies,^29^, ^30^ the luciferase report assay proved that Nrf2 was a certain target of miR‐144‐3p. Besides, overexpression or inhibition of miR‐144‐3p not only led to the changes of cell apoptosis, but also lead to changes in the expressions of Nrf2/HO‐1 pathway proteins and proteins related to the function of mitochondria and endoplasmic reticulum(GRP78, CHOP, Cyt C). Importantly, the inhibitory effect of miR‐144‐3p inhibitor on apoptosis and mitochondria or endoplasmic reticulum were apparently rescued when si‐Nrf2 was simultaneously transfected, which further confirmed that Nrf2 was a direct downstream target of miR‐144‐3p. Besides, the function of mitochondria and endoplasmic reticulum was closely related to miR‐144‐3p/Nrf2/HO‐1 pathway. Similar findings were reported in a model of ischaemia/reperfusion‐induced neuronal injury.^39^

LncRNAs are a set of RNA transcripts of more than 200 nucleotides which can influence posttranscriptional regulation through molecular sponge effects to silence miRNA expression, thus reducing miRNA‐mediated suppression of its target genes.^40^, ^41^, ^42^ Accumulating studies have revealed the important regulatory roles of lncRNAs in renal IRI including TUG1.^43^, ^44^, ^45^ TUG1 was recently reported to play important roles in ischaemia‐induced oxidative damage including in kidney, cardiac or cerebrum.^14^, ^46^, ^47^ MiR‐144‐3p was confirmed to be a target of TUG1 in tumour models,^18^, ^19^ however, among all these studies related to oxidative damage, miR‐144‐3p was never studied as a target of TUG1. Our results show that the expression of lncRNA‐TUG1 is significantly upregulated in renal IRI, and we also confirmed that TUG1 can directly bind to miR‐144‐3p, and their expression levels are negatively correlated. According to our present study, the overexpression of TUG1 in vivo or in vitro apparently decreased the expressions of miR‐144‐3p and reduced cell apoptosis via activating the Nrf2/HO‐1 pathway and the protective role of mitochondria or endoplasmic reticulum. However, this anti‐apoptosis effect was reversed when miR‐144‐3p mimics or si‐Nrf2 was co‐transfected. On the contrary, the inhibition of TUG1 obviously increased the expressions of miR‐144‐3p and accelerated cell apoptosis through inhibiting the Nrf2/HO‐1 pathway and the physiological function of mitochondria or endoplasmic reticulum. However, this proapoptotic effect was rescued when miR‐144‐3p inhibitor was co‐transfected, but reversed again when si‐Nrf2 was simultaneously transfected. Our results demonstrated that TUG1 activates the Nrf2/HO‐1 pathway by sponging miR‐144‐3p to play a protective role in renal IRI.

It is important to note that we are not the first to investigate the role of TUG1 in renal IRI, previous two studies had recently studied the effect of TUG1 in regulating renal IR‐induced apoptosis or inflammation.^14^, ^48^ Interestingly, the results and the conclusions seem inconsistent which could be possibly induced by the differences of animal species, different ischaemia or reperfusion time or differentially used reagents and so on. Besides, it is widely believed that one lncRNA may have multiple interacting miRNAs, and interacting on different miRNAs may conduct completely opposite effects. Therefore, studies on different targets (miRNAs) of TUG1 may lead to different conclusions. On the contrary, our study is generally convincing and many reports support our findings and the final conclusion. TUG1 was previously reported to play protective roles in regulating mitochondrial bioenergetics in diabetic nephropathy,^49^, ^50^ and mitochondria were commonly regarded as a key organelle in mediating ischaemia/reperfusion injury‐induced cell apoptosis,^51^ which indicated that TUG1 may play protective roles through regulating mitochondrial bioenergetics in renal IRI. In addition, TUG1 was also confirmed to have connections with Nrf2 and protect tissues from oxidative damage.^52^, ^53^ Meanwhile, the connections between TUG1 and miR‐144‐3p or miR‐144‐3p and Nrf2 have been fully discussed above. At last, only male mice were included in our study and the lack of inclusion of female mice might be a limitation of our research.^54^

**FIGURE 11:**
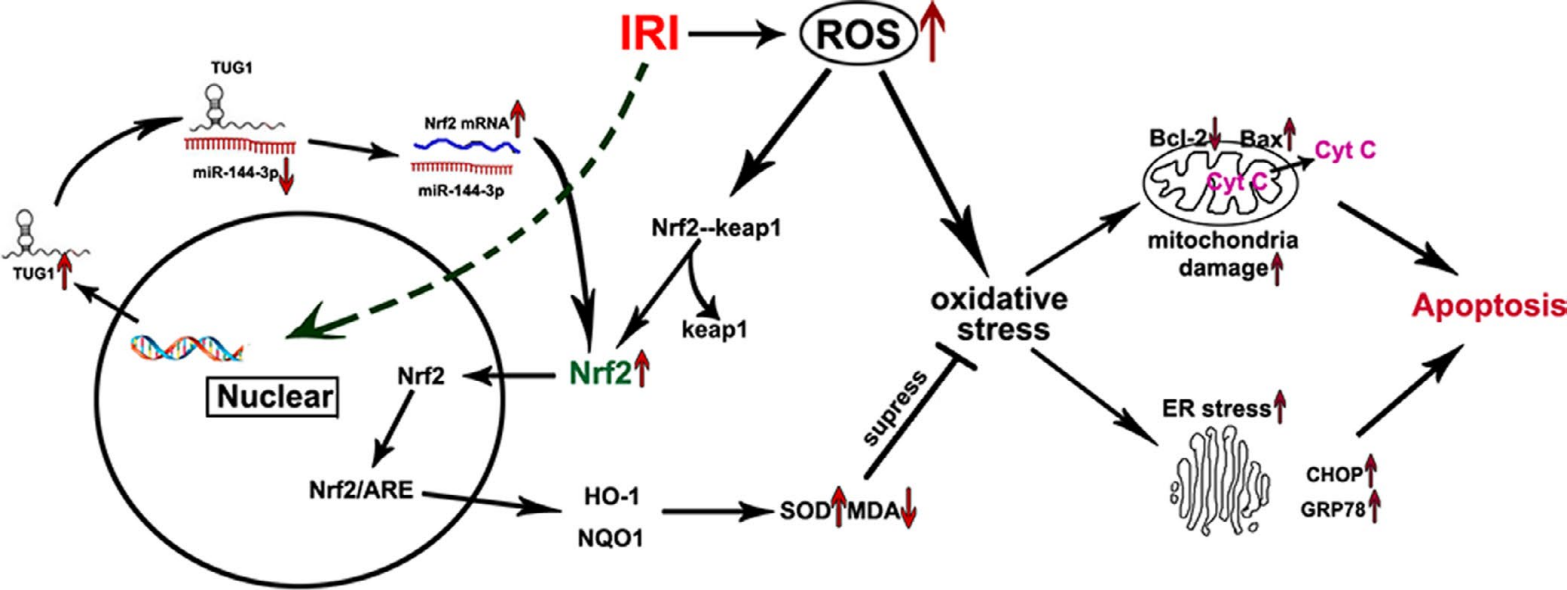
Schematic illustrations explains the signal transduction pathway. In short, the TUG1‐miR‐144‐3p‐Nrf2 axis regulates ischemia reperfusion‐induced cell apoptosis by adjusting oxidative stress, mitochondrial damage and endoplasmic reticulum stress

In conclusion, our present study firstly demonstrates that TUG1 plays a protective role in renal IRI by sponging miR‐144‐3p, activating the Nrf2/HO‐1 pathway, improving oxidative stress, mitochondrial damage and endoplasmic reticulum stress, which eventually attenuates apoptosis of renal tubular epithelial cells (Figure 11). This study provides new insights into the molecular mechanism of renal IRI, and the TUG1/miR‐144‐3p/Nrf2/HO‐1 pathway axis may be a promising new therapeutic target for renal IRI.

## CONFLICTS OF INTEREST

We declare that we have no commercial or associative interests that conflict with the work submitted.

## AUTHOR CONTRIBUTIONS

**Sheng Zhao:** Data curation (equal); Writing‐original draft (equal). **Wu Chen:** Data curation (equal); Formal analysis (equal); Methodology (equal); Writing‐original draft (equal). **Wei Li:** Data curation (supporting); Software (supporting). **Weimin Yu:** Investigation (supporting). **Siqi Li:** Formal analysis (supporting). **Ting Rao:** Investigation (supporting). **Yuan Ruan:** Software (supporting). **Xiangjun Zhou:** Validation (supporting). **Cong Liu:** Supervision (supporting). **Yucheng Qi:** Software (supporting). **Fan Cheng:** Funding acquisition (lead); Writing‐review & editing (lead).

## Data Availability

The data supporting the conclusions of this study can be obtained from the corresponding author.
